# SPOP regulates the expression profiles and alternative splicing events in human hepatocytes

**DOI:** 10.1515/biol-2022-0755

**Published:** 2023-10-24

**Authors:** Jing Dai, Xiang Dong, Yuxin Chen, Wanying Xue, Qingqing Wang, Feifei Shang, Yunxia Zhao, Shujing Li, Yu Gao, Yuanyuan Wang

**Affiliations:** School of Life Science, Bengbu Medical College, No. 2600 Donghai Road, Bengbu, China; Research Center of Clinical Laboratory Science, School of Laboratory Medicine, Bengbu Medical College, Bengbu, China; Department of Basic Medical College, Bengbu Medical College, Bengbu, Anhui, China; Anhui Province Key Laboratory of Translational Cancer Research, Bengbu Medical College, No. 2600 Donghai Road, Bengbu, Anhui, China

**Keywords:** speckle type BTB/POZ protein, gene expression profiles, alternative splicing events, human hepatocytes, hepatocellular carcinoma

## Abstract

Speckle type BTB/POZ protein (SPOP) may have cancer promoting or inhibiting effects. At present, the role of SPOP in hepatocellular carcinoma (HCC) has rarely been studied. In this study, to investigate the effects of SPOP in HCC and elucidate the underlying molecular mechanisms of its relationship with genes, differentially expressed genes (DEGs) were classified through RNA sequencing. The gene ontology analysis and Kyoto Encyclopedia of Genes and Genomes functional pathway analysis were used to further predict the function of DEGs after the overexpression of SPOP. The biological function of SPOP-regulated alternative splicing events in cells is comprehensively assessed. The Cancer Genome Atlas database and Gene Expression Omnibus dataset were performed to evaluate the correlation between SPOP and HCC progression. Due to SPOP overexpression, 56 DEGs in the HCC related pathway were further identified. The results showed that SPOP overexpression facilitated the cell proliferation and changed the gene expression profiles of human normal hepatocytes. SPOP-regulated alternative splicing events were involved in pathways associated with cellular processes, metabolism, environmental information procession, organismal systems, and so on. In conclusion, SPOP may potentially exhibit tumor-promoting effects, necessitating further investigations to unveil its molecular mechanisms comprehensively.

## 1 Introduction

Hepatocellular carcinoma (HCC) stands as the most prevalent form of primary liver cancer and ranks as the fourth leading cause of cancer-related deaths globally [1]. Up to now, the exact molecular mechanism in the progression of HCC is not completely clear. Clinical data and laboratory research results show that external environmental factors such as virus infection, alcoholism, and nutritional disorders may associate with the occurrence, development, deterioration, and metastasis of HCC [2–4]. At the same time, the genetic background of the individual also plays an important role during HCC carcinogenesis [5].

The occurrence of cancer may be related to the abnormal function of signal transduction pathways during cell division and proliferation [6]. Abnormal signal transduction may lead to abnormal gene regulation, leading to uncontrolled cell proliferation [7,8]. The ubiquitination level of various signal factors in cellular signal transduction pathways and networks is closely related to their content and activity. Ubiquitination constitutes a significant post-translational modification of eukaryotic proteins, enabling an important pathway for the non-lysosomal degradation of proteins [9,10]. The dynamic balance of protein ubiquitination and deubiquitination plays an important role in maintaining the stability of individual genomes and regulating the process of tumorigenesis [11].

Speckle type BTB/POZ protein (SPOP) derives its name primarily from its nuclear distribution and the presence of a POZ domain [12]. The SPOP gene is located at human 17q21.33, with a coding region of 1,125 bp, encoding 374 amino acids, with an approximate molecular weight of 42 kDa [13]. The protein of SPOP contains an N-terminal MATH region, a BTB/POZ region, a 3-box region, and a C-terminal NLS region [14]. Notably, the MATH region predominantly governs substrate recognition and binding, while the POZ region chiefly interacts with the E3 ligase complex [15]. Being an aptamer of the E3 ubiquitin ligase complex, SPOP is a very crucial element in the ubiquitination of various substrate proteins [16]. In various cancer cell types and under differing expression conditions SPOP may play different roles and mechanisms. For example, SPOP can inhibit the growth rate of cancer cells in lung cancer, colorectal cancer, prostate cancer, and so on. However, SPOP might also play a role in promoting cancer. It was reported that SPOP could promote the epithelial–mesenchymal transition of kidney, epithelial–mesenchymal transition in renal cancer epithelial cells, and promote the development of renal cancer [17]. The role of SPOP in HCC is rarely studied, only Huang’s team reported that SPOP can inhibit the proliferation of HCC cells and suppress the metastasis and mesenchymalization of HCC cells by downregulating the mRNA transcription level of Zeb2 [18].

SPOP exhibits a dual role, both as a tumor promoter and a tumor suppressor, although the research about its specific mechanism is still limited. As ongoing research advances the mechanism of SPOP function in different tumors might become clearer. The main purpose of this study was to investigate the downstream signaling pathways and the corresponding molecular mechanisms of SPOP in HCC.

## 2 Materials and methods

### 2.1 Cell culture and lentivirus transfection

Human normal hepatocytes WRL68 (ATCC, Manassas, USA) were cultured in Dulbecco’s Modified Eagle Medium (cat. no. PM150210, Procell, Wuhan, China) with 10% Gibco™ fetal bovine serum (cat. no. 10099141C, Invitrogen, AUS) and 1% penicillin–streptomycin (10,000 U/mL) (cat. no. 15140122, Invitrogen, USA), at 37°C in a 5% CO_2_ humidified atmosphere. To establish the SPOP overexpression model, lentivirus containing the SPOP gene was constructed and packaged by GeneCopoeia (Guangzhou, China). SPOP-overexpressing lentivirus was transfected into WRL68 cells (the SPOP group), resulting in the formation of the SPOP group. In parallel, an empty lentivirus was transfected into WRL68 cells to serve as the control group (referred to as the vec group). The transfection flow was performed according to the reagent manufacturer’s instructions using the Lipofectamine™ 2000 Transfection Reagent (cat. no. 11668027, Invitrogen, USA). The cells were selected with 1.5 μg/mL puromycin for 2 weeks.

### 2.2 Assessment of SPOP gene overexpression

Total RNA was extracted from the SPOP group and the vec group cells, respectively, using the RNA isolater Total RNA Extraction Reagent (cat. no. R401-01, Vazyme, China). Subsequently, the cDNA was synthesized according to the reverse transcription instruction of Hiscript III Reverse Transcriptase (cat. no. R302-01, Vazyme, China). The real-time fluorescent quantitative PCR (qRT-PCR) was conducted using Taq Pro Universal SYBR qPCR Master Mix (cat. no. Q712-02, Vazyme, China) in LightCycler^®^ 96SW1.1 real-time PCR system (Roche). Glyceraldehyde-3-phosphate dehydrogenase (GAPDH) was selected as the mRNA internal reference to access the efficiency of the SPOP overexpression. The primer sequences are listed in Table 1. The expression levels of SPOP in different groups were normalized to GAPDH mRNA levels using the 2^−ΔΔCT^ method.

**Table 1:** Primer sequences for qRT-PCR

Gene name	Forward primer sequence (5′−3′)	Reverse primer sequence (5′−3′)
GAPDH	ATGGGTGTGAACCATGAGAAGTA	GAGTGGGTGTCGCTGTTGAAGTC
SPOP	GCCCCGTAGCTGAGAGTTG	ACTCGCAAACACCATTTCAGT
CALU	AATAGACGCGGATAAAGATGGGT	GCCATTGGTTTTCAACATTGTCA
CCNA1	GAGGTCCCGATGCTTGTCAG	GTTAGCAGCCCTAGCACTGTC
CCNA2	CGCTGGCGGTACTGAAGTC	GAGGAACGGTGACATGCTCAT
CCNC	CCTTGCATGGAGGATAGTGAATG	AAGGAGGATACAGTAGGCAAAGA
CDK1	GGATGTGCTTATGCAGGATTCC	CATGTACTGACCAGGAGGGATAG
CDK4	ATGGCTACCTCTCGATATGAGC	CATTGGGGACTCTCACACTCT
CDK6	CCAGATGGCTCTAACCTCAGT	AACTTCCACGAAAAAGAGGCTT
FXR1	CTGCGACAGATTGGTTCTAGG	TGTACCATAACCGGAGGTGTAA
GIGYF1	AACCTGCTCCCGACGATGA	AGGCTGAGGTATGTACGTCCC
KIAA1522	CCAGGACAACGTCTTCTTTCC	CAGCCACCCTTGTTCAGTTTC
KRAS	ACAGAGAGTGGAGGATGCTTT	ACAGAGAGTGGAGGATGCTTT
MYBL2	CCGGAGCAGAGGGATAGCA	CAGTGCGGTTAGGGAAGTGG
PAPOLG	TGTCTCTGGATAGCAGTTGTCTGG	TTCGTCCTACTACGGTAGGAATGG
PER1	GCCAACCAGGAATACTACCAGC	GTGTGTACTCAGACGTGATGTG
PRKAG2	TGCCCGTTATTGACCCTATCA	CAGGCTTTGGCATATCAGACAT
SOX12	AAGAGGCCGATGAACGCATT	TAGTCCGGGTAATCCGCCAT
TP53	CAGCACATGACGGAGGTTGT	TCATCCAAATACTCCACACGC
XPO1	ATCTGACCCAACTTGTGTAGAGA	TGGTCCTACTTGCTCCAACAAT

### 2.3 Cell counting kit 8 (CCK8) assay

Cell proliferation was tested using CCK8 (cat. no. C0038, Beyotime Biotechnology, China). Cells from different groups were seeded at a density of 1 × 10^3^ cells in 100 μL culture medium per 96-well plates for cell culture for 24, 48, 72, and 96 h. Then, CCK8 reagent was added with 10 μL to each well. After incubation at 37°C for 2 h, the absorbance at 450 nm (OD) of each well was measured.

### 2.4 Colony formation assay

During colony formation assay, cells were seeded at a density of 1 × 10^3^ cells/well into six-well plates, and cultured for 2 weeks. Two weeks later, colonies were fixed in 4% paraformaldehyde for 10 min, stained with 0.5% crystal violet for 10 min, and gently rinsed three times with PBS. A spot with more than 50 cells was counted as one clone.

### 2.5 RNA sequencing

The RNA samples of the SPOP group and the vec group cells were sequenced with three replicates per group at Novogene Co., Ltd (Beijing, China). RNA was extracted from cells using TRIzol reagent, followed by strict quality control of RNA samples using RNA Nano 6000 Assay Kit of the Bioanalyzer 2100 system. For library preparation, the NEBNext^®^ Ultra™ Directional RNA Library Prep Kit for Illumina^®^ (NEB, USA) was used. The insert size of the resulting library was assessed using the Agilent 2100 Bioanalyzer. After library preparation, the samples were subjected to sequencing on an Illumina platform, which generated 150 bp paired-end reads. The paired-end clean reads were aligned to the human reference genome using the software HISAT2 [19]. The data files for RNA-seq have been deposited in the Sequence Read Archive database under PRJNA853057.

### 2.6 Screening of differentially expressed genes (DEGs)

After processing and analysis of RNA sequencing data, the alignment results were transferred to the program StringTie for transcript assembly [20]. The gene expression levels of RNA-seq were expressed as fragments per kilobase of transcript sequence per million base pairs sequenced (FPKM). To identify DEGs between the SPOP group and the vec group, the DESeq algorithm, implemented as a package in the R software, was utilized. The resulting *p*-values were adjusted using the Benjamini and Hochberg’s approach for controlling the false discovery rate. The thresholds to screen DEGs were |log2Fold change (FC)| > 0 and adjust *p*-value <0.05.

### 2.7 qRT-PCR validation of selected DEGs

To verify the validity of the RNA-seq results, qRT-PCR was conducted on some randomly selected DEGs, which were identified using RNA-sequencing. The primer sequences used for DEGs validation are also presented in Table 1. During the validation process, the RNA expression levels of the selected genes were standardized with that of GAPDH.

### 2.8 Gene function enrichment analysis

The gene ontology (GO) analysis and Kyoto Encyclopedia of Genes and Genomes (KEGG) functional pathway analysis were used to predict the function of DEGs between the SPOP group and the vec group. Additionally, the DisGeNET databases were also used to screen coronary liver disease-related disease targets. The enrichment was regarded as be significant when the corrected *p*-value was less than 0.05.

### 2.9 Alternative splicing events (ASEs) analysis

The ASEs between the SPOP group and the vec group were defined and quantified using the rMATS software. In this study, according to the splice junction reads, five types of ASEs were identified, including alternative 3′ splice site (A3SS), alternative 5′ splice site (A5SS), mutually exclusive exons (MXE), retained intron (RI), and skipped exon (SE). To evaluate the significance of SPOP-regulated ASEs, a Student’s *t*-test was performed to evaluate the significance of the ratio alteration of ASEs. The thresholds to screen DEGs were adjust to a *p*-value <0.05.

### 2.10 Public data source bioinformatics analysis

HCC expression profile data were obtained from The Cancer Genome Atlas (TCGA) database and Gene Expression Omnibus (GEO) database. The TCGA database encompassed 374 HCC patients vs 50 normal control individuals and 50 pairs of HCC tissues vs matched adjacent non-tumor tissues. The relevant clinical data, including, age, sex, grade, invasion depth, lymph node metastasis, and distant metastasis were also obtained from TCGA database. Additionally, we retrieved raw-intensity expression files of GSE14520, GSE25097, and GSE45436 from GEO database. The SPOP expression levels in the paraneoplastic and HCC tissues of every patient were analyzed. The effect of SPOP expression levels on the survival of HCC patients was estimated through the Kaplan–Meier (KM) plotter (http://kmplot.com/analysis/), an online survival analysis tool.

### 2.11 Statistical analysis

All the above analyses were performed by using R software version 4.0.3. Differential gene expression analysis was performed using the limma package in R. To investigate the association between DEGs and survival outcomes, KM survival curves were generated using the ggsurvplot function from the survminer package. All experiments were repeated three times, and the data were expressed as mean ± standard deviation. *T*-test and paired *t*-test were used to analyze the differences between the two groups by independent samples. Pearson correlation test provided the basis for correlation analysis. The relationship between SPOP expression and clinical characters was performed using the Wilcoxon test. Statically significance was defined as *p* < 0.05 (*), *p* < 0.01 (**), and *p* < 0.001 (***).

## 3 Results

### 3.1 SPOP overexpression facilitated the proliferation of WRL68 human normal hepatocytes

**Figure 1:**
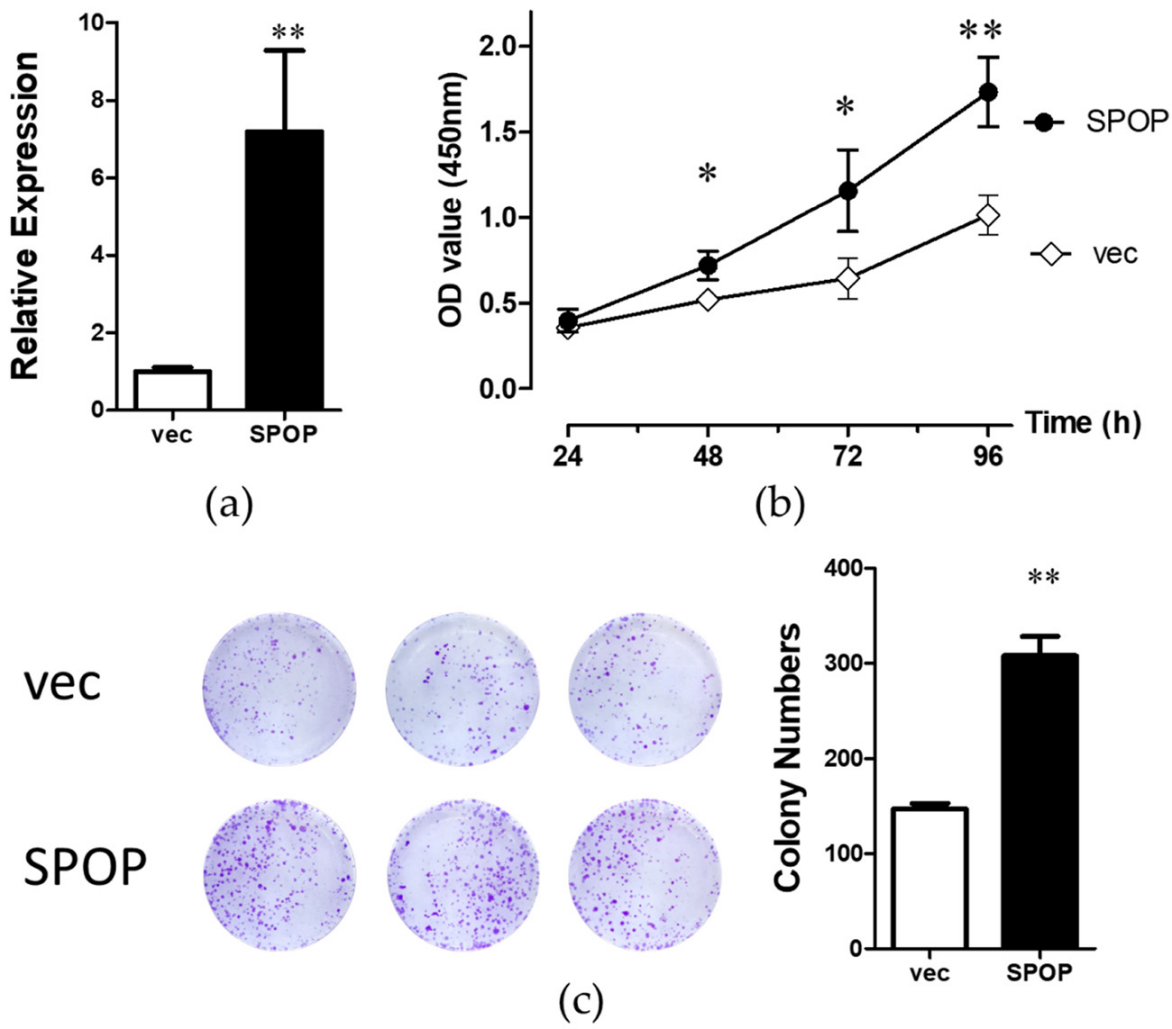
Overexpression of SPOP could facilitate the proliferation of human normal hepatocytes (WRL68). (a) Transfection efficiency of SPOP in WRL68 cells was measured by qRT-PCR. (b) Proliferative abilities of WRL68 cells in the SPOP group and the vec group were detected by CCK8 assay. (c) Colony formation assay detected the proliferations of WRL68 cells in the SPOP group and the vec group. *p < 0.05, **p < 0.01.

To investigate the influence of SPOP on the proliferation of human normal hepatocytes (WRL68), the SPOP overexpression model in WRL68 cells was constructed by transfection with SPOP-overexpressing lentivirus (the SPOP group). Simultaneously, the empty lentivirus was transfected into WRL68 cells as a control group (the vec group). qRT-PCR was operated to verify the transfection efficiency, which successfully induced the increased expression of SPOP in WRL68 cells (Figure 1a). The proliferation ability of the SPOP group was significantly higher than that of the vec group at each period of 48, 72, and 96 h (Figure 1b). The colony formation assay was also conducted to access cell proliferation. It turned out that there was a considerable increase of cell proliferation in the SPOP group compared with the vec group (Figure 1c, *p* < 0.05). These findings strongly suggest the SPOP overexpression and the proliferation ability of WRL68 cells.

### 3.2 SPOP overexpression changed the gene expression profiles of WRL68 human normal hepatocytes

**Figure 2:**
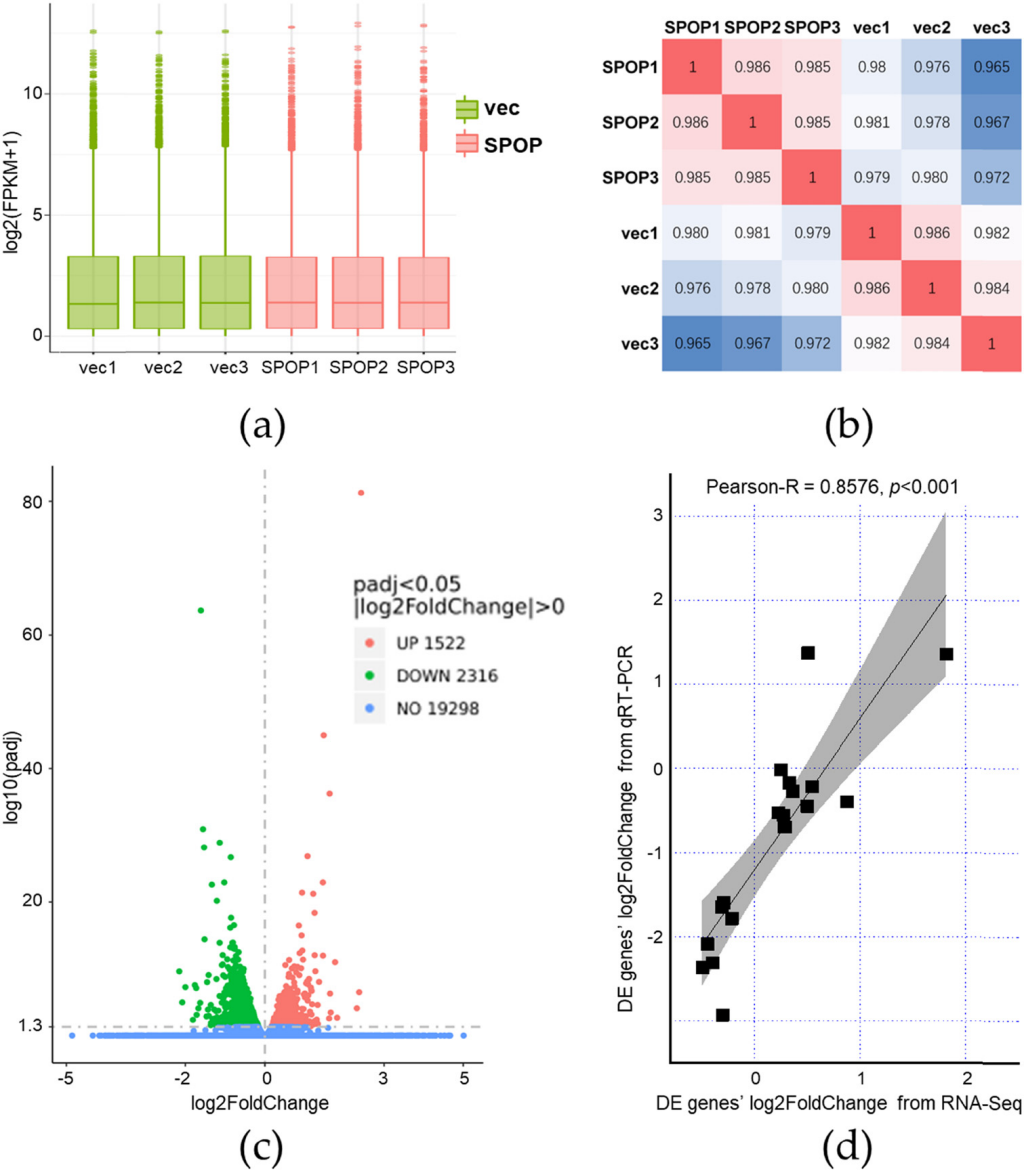
Overexpression of SPOP could change the gene expression profiles of WRL68. (a) Boxplot of sample gene expression distribution. (b) Correlation between SPOP overexpression (SPOP rep1/2/3) and control (vec rep1/2/3) samples. (c) DEGs between the SPOP group and the vec group. (d) Correlation of log2 fold-change between RNA-Seq and RT-qPCR for significantly DEGs.

To characterize the function of SPOP in human normal hepatocytes, RNA-seq of SPOP overexpressed WRL68 cells (the SPOP group) and control WRL68 cells (the vec group) was performed (PRJNA853057). The gene expression levels of RNA-seq were quantified in FPKM. The distribution of gene expression levels in each sample of the SPOP group and the vec group is shown as the boxplots (Figure 2a). Despite having only three biological replicates in each group, there was a significant correlation between the SPOP group and the vec group (Figure 2b). Genes with |log2 (Fold Change)| > 0 and *p*_adj_ <0.05 were assigned as DEGs, and there were 3,838 DEGs induced by SPOP overexpressing, including 1,522 upregulated genes and 2,316 downregulated genes (Figure 2c). The list of DEGs is presented in Table S1. To verify DEGs identified through from RNA-seq analysis, the gene expression levels of 18 genes were also measured by qRT-PCR. Between the two techniques, the Pearson’s correlation coefficient of fold change in gene expression levels was 0.8576 (*p <* 0.001) (Figure 2d).

### 3.3 Overview of affected biological functions in WRL68 human normal hepatocytes with SPOP overexpressing

To explore the potential biological functions of SPOP-related genes, we conducted GO, KEGG, and DisGeNET analyses using significant DEGs.

**Figure 3:**
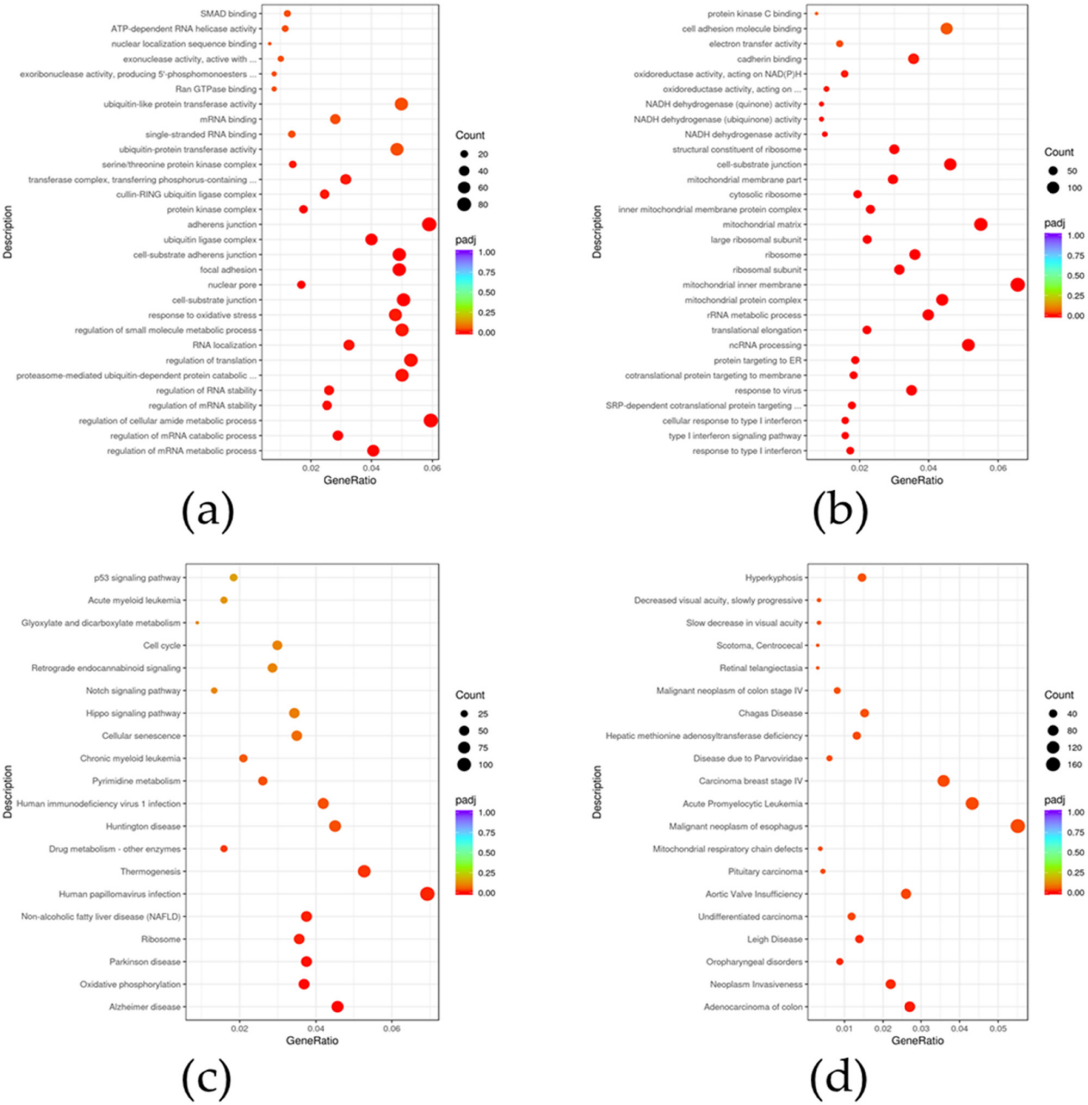
Potential biological functions of SPOP-related genes in WRL68 cells. (a) Top 30 most enriched GO terms of the upregulated DEGs. (b) Top 30 most enriched GO terms of the downregulated DEGs. (c) Top 20 most enriched KEGG pathways of all DEGs. (d) Top 20 most enriched DisGeNET lists of all DEGs.

For the upregulated genes, the enriched GO biological processes were mostly related to the regulation of mRNA metabolic process, mRNA stability, and translation (Figure 3a). The complete list is given in Table S2. Conversely, the GO biological processes enriched by the downregulated genes were mostly related to protein targeting and the response to type I interferon and virus, and so on (Figure 3b, Table S3). SPOP overexpression led to the identification of 11 KEGG pathways with *p*_adj_ <0.05, including oxidative phosphorylation, drug metabolism, pyrimidine metabolism, and so on (Figure 3c, Table S4). Since less than 20 enriched KEGG pathways reached significant according to adjusted *p*-values, the top 20 most enriched KEGG pathways of all DEGs are listed in Figure 3c based on *p*-values. With SPOP overexpressing, 25 DisGeNET with *p*_adj_ <0.05 were identified, including neoplasm invasiveness, undifferentiated carcinoma, hepatic methionine adenosyltransferase deficiency, steatohepatitis, and so on (Figure 3d, Table S5).

### 3.4 Analysis of potential SPOP‑regulated ASEs and enrichment biological function analysis

**Figure 4:**
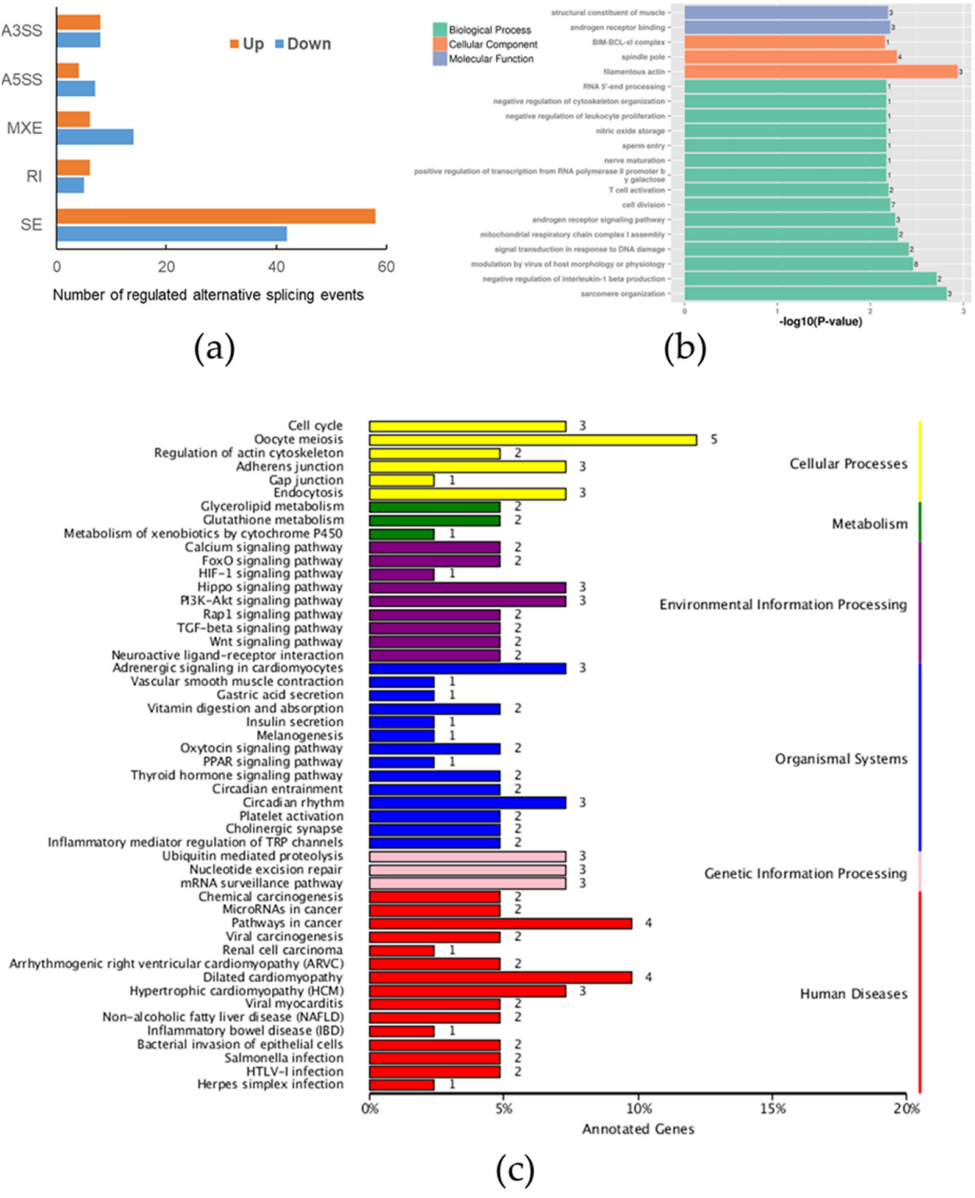
Enrichment biological function of SPOP RASEs in WRL68 cells. (a) Number of significant RASEs. (b) GO functional enrichment of different RASEs. (c) KEGG pathway enrichment analysis results: A3SS events, 11 A5SS events, 20 MXE events, 11 RI events, and 100 SE events.

To identify the regulatory effect of SPOP on alternative splicing, SPOP-affected ASEs were analyzed by using transcriptional sequencing data from WRL68 SPOP cells in the SPOP group and the vec group. This analysis identified a total of 158 RASEs that showed significant differences between the two groups. Of the RASE types, there were 16 A3SS events, 11 A5SS events, 20 MXE events, 11 RI events, and 100 SE events (Figure 4a). The complete list of RASEs is given in Table S6. When SPOP was overexpressed, the proportion of SE events was increased. The GO enrichment results revealed that SPOP-regulated ASEs were mainly enriched in modulation by virus of host morphology or physiology, cell division, sarcomere organization, and so on (Figure 4b). Furthermore, KEGG pathway enrichment showed that SPOP-regulated ASEs were involved in pathways associated with cellular processes, metabolism, environmental information procession, organismal systems, and more (Figure 4c).

### 3.5 Bioinformatics analysis of SPOP in HCC

**Figure 5:**
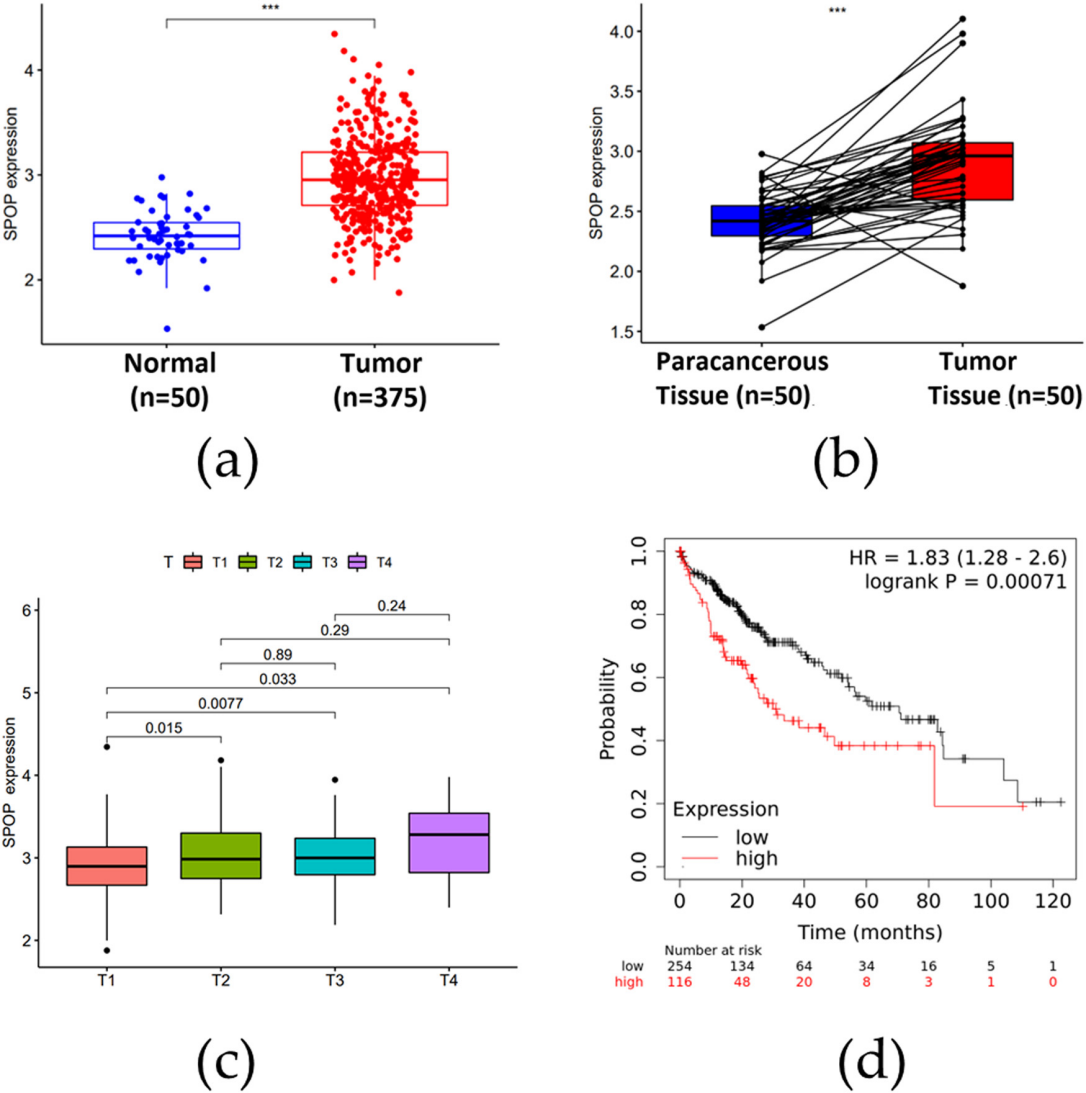
Results of bioinformatics analysis of SPOP in HCC. (a) Differential expression of SPOP in HCC tissues and normal liver tissues. (b) Differential expression of SPOP in paraneoplastic and HCC tissues. (c) Correlation of SPOP expression with tumor stage. (d) Analysis of overall survival of SPOP expression in HCC patients.

To explore the correlation between SPOP expression levels and the procession of HCC, the bioinformatics analysis of SPOP was carried out. According to the data from TCGA database, the significant upregulations of SPOP expression were observed in HCC tissues compared to normal liver tissues (Figure 5a). The expression of SPOP was also significantly higher in HCC tissues compared to paraneoplastic tissues (Figure 5b). The result of relationship between SPOP expression and clinicopathological parameters showed that SPOP expression was positively correlated with tumor stage (Figure 5c). According to the results of the KM mapper analysis, it was evident that the survival rate of SPOP high expression group was remarkably lower than that of low expression group (Figure 5d).

### 3.6 DEGs due to SPOP overexpression in the HCC-related pathway in WRL68 cells

**Figure 6:**
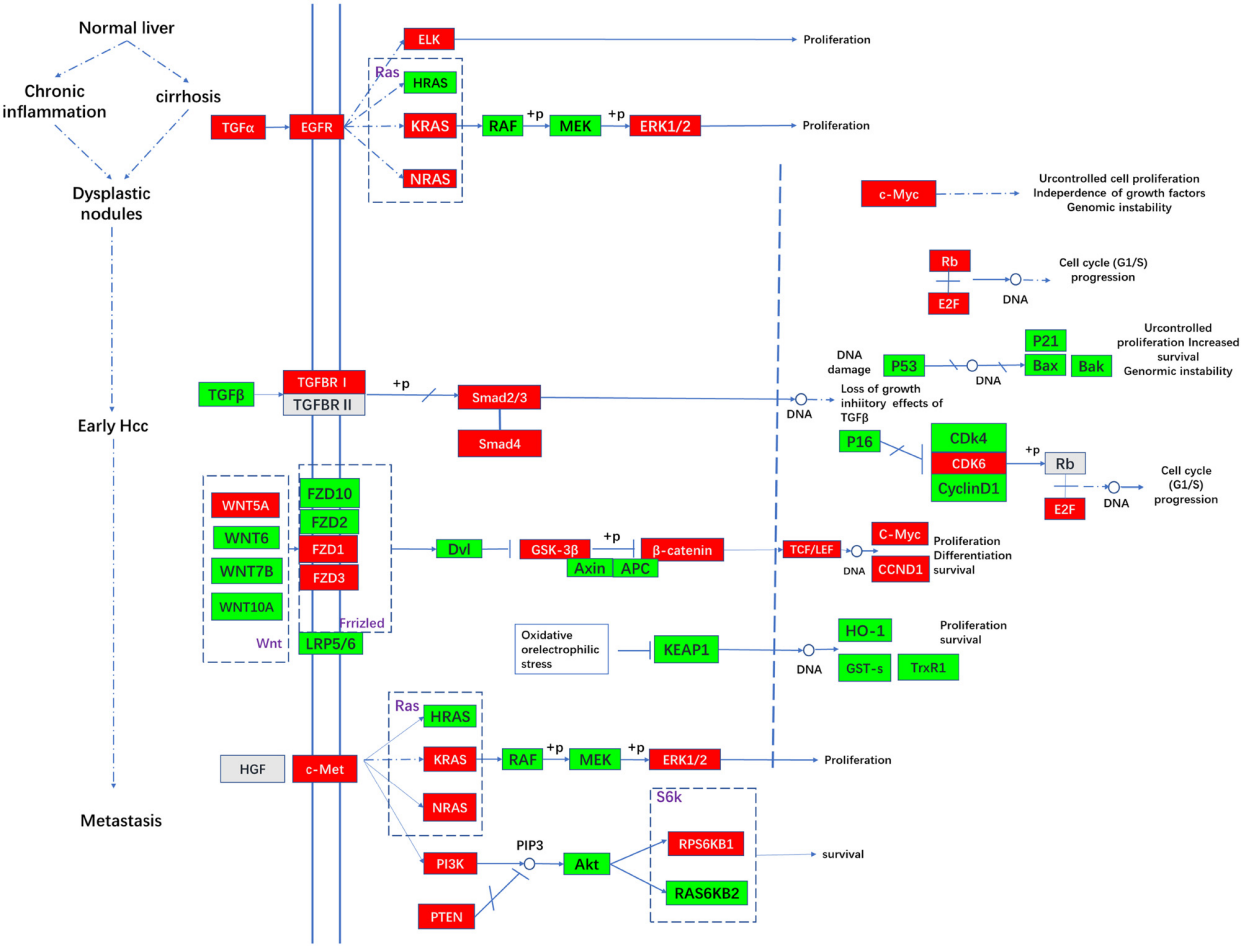
HCC pathway modified by SPOP overexpression in WRL68 (the figure was drawn according to the information from KEGG PATHWAY database. The molecules in red were upregulated with SPOP overexpression and the molecules in green were downregulated with SPOP overexpression).

The result of bioinformatics analysis showed that of SPOP might play a role in procession of HCC. Due to SPOP overexpression, a total of 56 DEGs were implicated in the HCC pathway, among which 33 were downregulated and 23 were upregulated. In Figure 6, genes colored in red represent upregulated expression in WRL68 cells with SPOP overexpression compared to cells in the control group, while genes colored in green indicate downregulated expression. From the status of the normal liver to dysplastic nodules, nine were downregulated and only four were upregulated. From the status of dysplastic nodules to early HCC, nine were downregulated and only four were upregulated. In the process of HCC metastasis, approximately nine genes were downregulated and four were upregulated. The proteins encoded by DEGs were located in different parts of the cells. About four down- and two upregulated genes were located outside, and three down- and five upregulated genes were located on the cell membrane. Moreover, nine down- and eight upregulated genes were located in the nucleus.

## 4 Discussion

The development of HCC is a complex process influenced by both environmental and genetic factors [21,22]. It is well known that the ubiquitin proteasome pathway could play an important role in the progression of cirrhosis and HCC [23]. Ubiquitination serves as a crucial post-transcriptional modification of eukaryotic proteins and represents an important pathway for non-lysosomal degradation of proteins [24]. SPOP, as an important protein molecule involved in the regulation of the occurrence and development of various tumors, has limited reports regarding its molecular mechanism in liver cancer metastasis [25]. For instance, Huang’s team reported that SPOP could inhibit hepatoma cell migration, including the suppression of ZEB2 expression and the related program of epithelial–mesenchymal transition [18]. The results from Ji’s group showed that SPOP expression was downregulated in HCC and also related to tumor size, differentiation, and metastasis [26]. However, according to the TCGA data, SPOP was upregulated markedly in HCC tissues compared to normal liver tissues. The expression of SPOP was also significantly higher in HCC tissues compared to paraneoplastic tissues. SPOP expression was upregulated in HHC patients, which is significantly related to lower survival rate and poor prognosis. Collectively, all of these indicated that SPOP has a tumor-promoting effect in HCC, prompting us to explore its impact further. Despite limited knowledge about the transcriptional levels of SPOP in human normal hepatocytes, our bioinformatics analysis which involved SPOP overexpression in WRL68 showed that SPOP regulates the expression profiles and ASEs in human hepatocytes.

SPOP, a crucial ubiquitinylation ligase, also serves as a bridging component of Cul3 (a member of the E3 family of ubiquitin ligases) which binds to substrate proteins. SPOP is involved in the regulation of a variety of cellular activities, influencing two major categories of fundamental cellular activities including genome modification and cellular signaling. In these processes, SPOP mediates ubiquitination modifications of many nuclear proteins, leading to protein degradation and thus regulating a variety of cellular functions. In this study, we aimed to uncover the mechanism through which SPOP is involved in human hepatocytes. To achieve this, we established a cell model with SPOP overexpressed. A total of 3,838 DEGs were reliably detected by transcriptome profile, including 1,522 upregulated genes and 2,316 downregulated genes. The enrichment of gene function indicated that the upregulated genes were mostly related to the regulation of mRNA metabolic process, mRNA stability, and translation, while the downregulated genes were mostly related to response to type I interferon, response to virus, and protein targeting. Moreover, RASEs were identified with SPOP overexpression.

In this study, a total of 56 DEGs were identified in the pathway of HCC with SPOP overexpression. During the early stage of HCC development, the reduced expression of transforming growth factor-beta (TGF-β) emerges as a crucial factor [27,28]. It has been reported that TGF-β plays a vital role in cell proliferation and differentiation [29–31]. In response to TGF-β-mediated dimerization between TGF-β receptor I (TGF-βRI) and TGF-βRII, receptor-regulated SMADs (SMAD2/3) are phosphorylated, interact with SMAD4, and translocate to the nucleus with some DNA-binding partners and transcriptional co-activators or co-repressors to form a complex controlling gene transcription [32]. TGF-β/SMAD3 signaling pathway plays a critical role in inhibiting tumor formation by suppressing cell growth and promoting apoptosis, both in normal cells and during the early stages of tumorigenesis. Jiao et al. reveals that SPOP expression is repressed by TGF-β/SMAD signaling axis in PCa CSCs [29]. Our data demonstrated that overexpressed SPOP in human normal hepatocytes WRL68 decreased the mRNA level of TGF-β in a novel TGF-β/SMAD3 signaling pathway. Downregulated TGF-β may play a role in promoting cell proliferation and tumor formation in WRL68. In this study, SPOP expression may negatively regulate SMAD3-mediated TGF-β signaling pathway in human normal hepatocytes. Moreover, during the early HCC, the expression levels of certain genes involved in the PI3K-Akt signaling pathway, p53 signaling pathway, and Wnt signaling were also regulated with SPOP overexpression. These gene alterations may indeed affect various aspects of cellular function, including cell proliferation, cell cycle progression, telomeric DNA repeats, maintenance of telomere ends, DNA damage, genomic instability, and so on.

During the metastasis stage, the cellular-mesenchymal epithelial transition factor (c-Met) might play the key role [33]. The protein of c-Met is implicated in the central carbon metabolism in various cancer types, including renal cell carcinoma, melanoma, HCC, gastric cancer, non-small cell lung cancer, and so on [34]. The overexpression of SPOP was found to trigger a cascade phosphorylation reaction of c-Met, which in turn amplified the signaling process, ultimately leading to the activation of various downstream pathways such as PI3K/Akt and RAS/MAPK. These activated signaling pathways collectively regulated a range of biological processes, including cell growth, survival, motility, and proliferation. The abnormal c-Met signaling pathway has been reported in a variety of tumor studies. Normally, the c-Met signaling pathway is only fully activated during wound healing and tissue regeneration, but c-Met signaling pathway in tumor could be frequently activated by cancer cells, leading to tumor formation, invasive growth, and metastasis. The c-Met signaling pathway has been reported that it might be abnormally regulated in many types of solid tumors (lung, gastric, liver, colorectal, etc.) and could play a crucial role in the development of colorectal cancer, the invasion and metastasis of HCC, and the formation, growth and metastasis of oral squamous cancer [35].

RASEs are key biological processes that extend gene expression patterns and generate protein diversity [36]. Growing evidence indicates that protein heterodimers resulted by variable splicing are closely linked in the formation of many tumors, including HCC, and are valuable as next-generation diagnostic markers due to their specificity of expression. In this study, a total of 158 RASEs were identified with SPOP overexpressed. The enrichment analysis suggested that RASEs would modify the function via calcium, FoxO, HIF-1, Hippo, PI3K-Akt, Rap1, TGF-beta, and Wnt signaling pathways, which play key roles during environmental information processing. Moreover, the changes of transcripts might be related to viral carcinogenesis, non-alcoholics fatty liver disease, cancer development, and so on.

## 5 Conclusions

In conclusion, RNA sequencing analysis offered a novel idea into the molecular mechanisms of SPOP in HCC. We explored the regulatory mechanism of SPOP overexpression in human normal hepatocytes. The downstream signaling pathways and the corresponding molecular mechanisms of SPOP manifest the potential of its tumor-promoting effect in HCC, and thus SPOP may serve as a new opinion for underlying tumor-promoting effect in HCC. Further studies are needed to better reveal the molecular mechanism of SPOP, and the effect of SPOP in the progression of HCC will become clear.

## Supplementary Material

Supplementary Table S1

Supplementary Table S2

Supplementary Table S3

Supplementary Table S4

Supplementary Table S5

Supplementary Table S6

Supplementary Table S7

Supplementary Table S8

Supplementary Table S9
